# Methylation alteration of *SHANK1* as a predictive, diagnostic and prognostic biomarker for chronic lymphocytic leukemia

**DOI:** 10.18632/oncotarget.27080

**Published:** 2019-08-13

**Authors:** Eleonora Loi, Loredana Moi, Antonio Fadda, Giannina Satta, Mariagrazia Zucca, Sonia Sanna, Shadi Amini Nia, Giuseppina Cabras, Marina Padoan, Corrado Magnani, Lucia Miligi, Sara Piro, Davide Gentilini, Maria Grazia Ennas, Melissa C. Southey, Graham G. Giles, Nicole Wong Doo, Pierluigi Cocco, Patrizia Zavattari

**Affiliations:** ^1^Department of Biomedical Sciences, Unit of Biology and Genetics, University of Cagliari, Cagliari, Italy; ^2^Department of Medical Sciences and Public Health, Occupational Health Unit, University of Cagliari, Cagliari, Italy; ^3^Department of Biomedical Sciences, Cytomorphology Unit, University of Cagliari, Cagliari, Italy; ^4^Unit of Hematology, A. Businco Oncology Hospital, Cagliari, Italy; ^5^Department of Medical Sciences, Unit of Medical Statistics and Cancer Epidemiology, University of Eastern Piedmont, Novara, Italy; ^6^Institute of Oncology Studies and Prevention, Florence, Italy; ^7^Department of Brain and Behavioral Sciences, University of Pavia, Pavia, Italy; ^8^Bioinformatics and Statistical Genomics Unit, Istituto Auxologico Italiano IRCCS, Cusano Milanino, Milan, Italy; ^9^Precision Medicine, Monash University, Melbourne, Melbourne, Australia; ^10^Department of Clinical Pathology, The University of Melbourne, Melbourne, Australia; ^11^Cancer Epidemiology and Intelligence Division, Cancer Council Victoria, Melbourne, Australia; ^12^Centre for Epidemiology & Biostatistics, The University of Melbourne, Melbourne, Australia; ^13^Concord Hospital Clinical School, The University of Sydney, Sydney, Australia

**Keywords:** cancer methylation alteration, predictive biomarkers, diagnostic biomarkers, prognostic biomarkers, SHANK1

## Abstract

Chronic lymphocytic leukemia (CLL) is a clinically heterogeneous disease characterized by the clonal expansion of malignant B cells. To predict the clinical course of the disease, the identification of diagnostic biomarkers is urgently needed. Aberrant methylation patterns may predict CLL development and its course, being very early changes during carcinogenesis. Our aim was to identify CLL specific methylation patterns and to evaluate whether methylation aberrations in selected genes are associated with changes in gene expression. Here, by performing a genome-wide methylation analysis, we identified several CLL-specific methylation alterations. We focused on the most altered one, at a CpG island located in the body of *SHANK1* gene, in our CLL cases compared to healthy controls. This methylation alteration was successfully validated in a larger cohort including 139 CLL and 20 control *in silico* samples. We also found a positive correlation between *SHANK1* methylation level and absolute lymphocyte count, in particular CD19+ B cells, in CLL patients. Moreover, we were able to detect gains of methylation at *SHANK1* in blood samples collected years prior to diagnosis. Overall, our results suggest methylation alteration at this *SHANK1* CpG island as a biomarker for risk and diagnosis of CLL, and also in the personalized quantification of tumor aggressiveness.

## INTRODUCTION

Chronic lymphocytic leukemia (CLL), one of the most common mature B cell neoplasm subtypes, is characterized by the monoclonal expansion of malignant B cells. CLL is a clinically and biologically heterogeneous disease. In fact, while some patients exhibit slow progression of the disease, others experience a more aggressive form and require adequate therapy to be initiated soon after diagnosis. No screening tests are currently recommended for this disease, and CLL is often diagnosed when undergoing blood tests for other reasons. The two staging systems currently used, Rai and Binet, remain good indicators of survival, but do not take into account other biological factors implied in the disease course, and consequently do not allow early recognition of the aggressive forms of the disease [1]. To date, several recurrent genomic aberrations, such as del(17p), del(11q), trisomy 12, and del(13q), and genetic alterations, such as *TP53*, *BIRC3*, *NOTCH1*, *SF3B1*, have been proposed as prognostic biomarkers of CLL cases [2], and their characterization is recommended before initiating therapeutic treatment. However, since genetic aberrations might not be present at diagnosis, and they might occur later during the disease course, their characterization is not always sufficient to predict the clinical outcome in the initial stage. The mutational status of *IGHV* gene is one of the most commonly used prognostic biomarkers but, alone, it is inadequate to explain the clinical course heterogeneity of this disease. Therefore, diagnostic biomarkers and prognostic biomarkers characterizing different clinical courses in the early stages of the disease are urgently needed.

Aberrant methylation patterns, which represent a molecular hallmark of cancer, are early events in tumor development, and they might represent early diagnostic biomarkers. It has been shown that methylome alterations would predict the diagnosis several years prior to the clinical appearance of the disease, thus proving to be potential markers for carrying out large-scale screening tests [3, 4]. Besides, aberrant methylation changes might represent a specific signature of different types of leukemia, depending on the originating cell [5]. Our aim was to identify specific-CLL methylation patterns by conducting a genome-wide methylation analysis, and to evaluate whether methylation aberrations in selected genes are associated with changes in gene expression.

## RESULTS

### Differential methylation analyses

**Figure 1:**
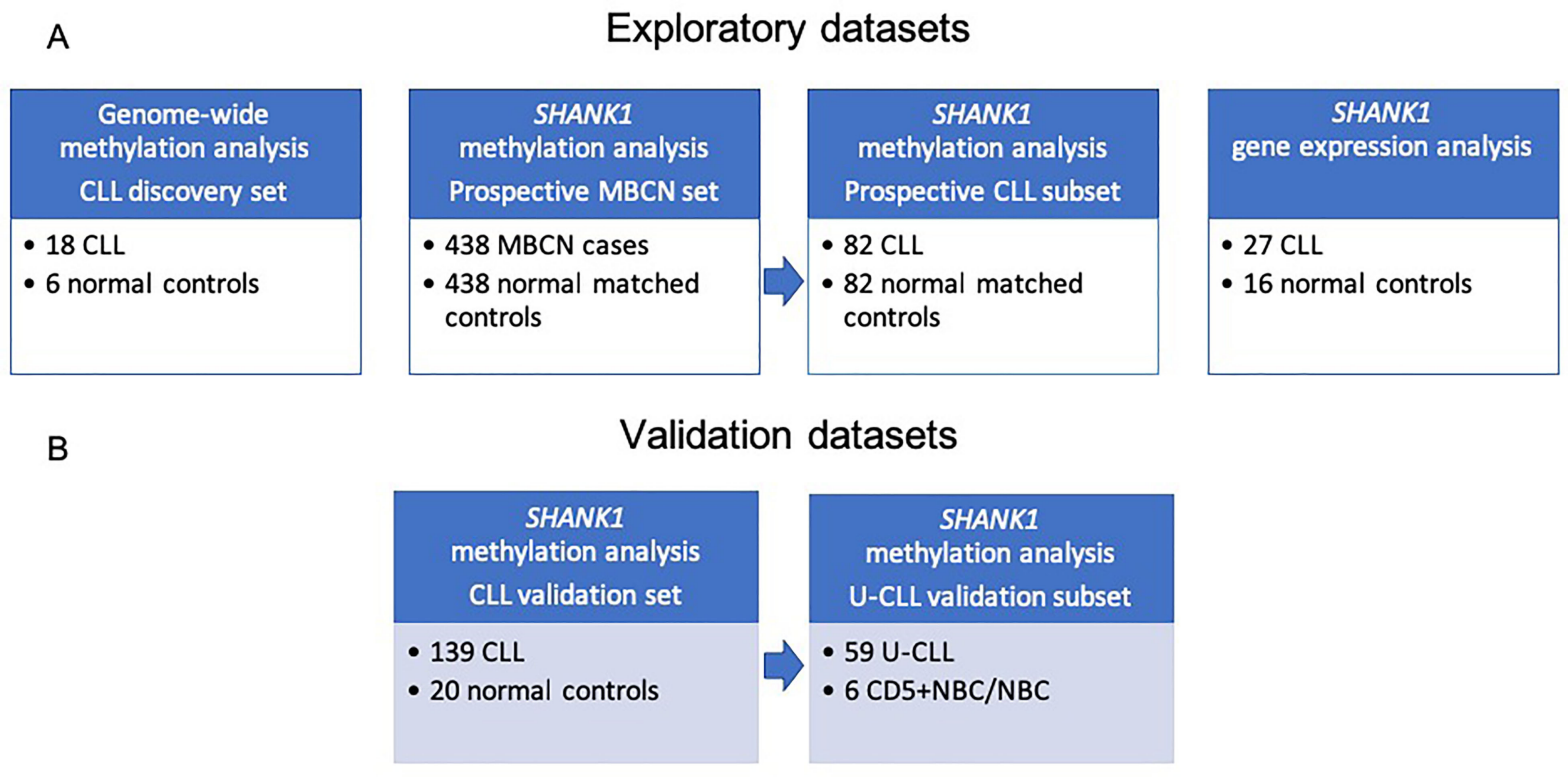
Study workflow including description of sample datasets used in the current study. **(A)** Discovery datasets: CLL, MBCN and normal samples for methylation and gene expression studies; **(B)** Validation datasets: CLL and normal samples analyzed to validate our finding. Abbreviations: CLL: chronic lymphocytic leukemia; MBCN: mature B-cell neoplasm; U-CLL: unmutated chronic lymphocytic leukemia.

**Figure 2:**
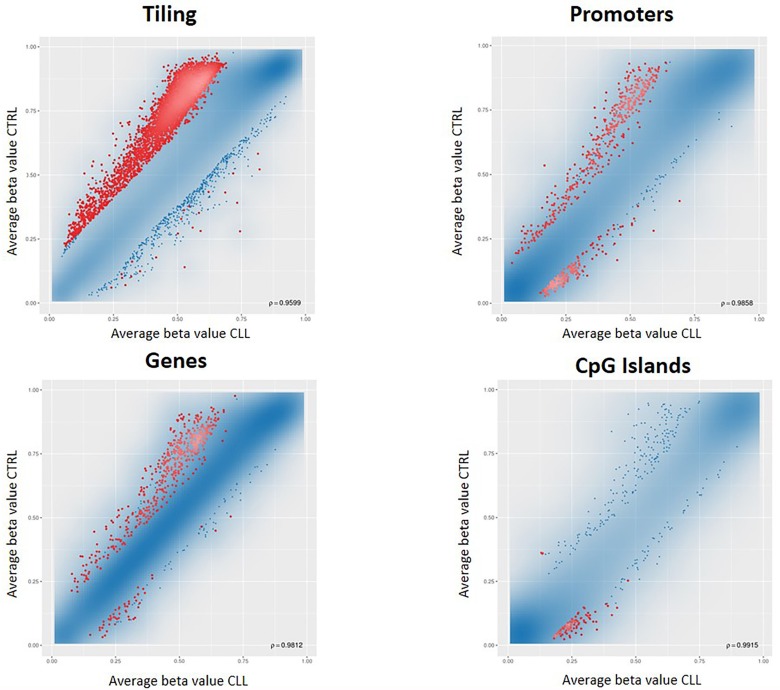
RnBeads differential methylation analysis for tiling, genes, promoters and CpG islands. Each dot represents the average beta value for each CpG locus in the region, resulting from the average of the samples belonging to that group. Red dots indicate CpG loci significantly differentially methylated between the two groups.

We conducted a first differential methylation analysis between 18 CLL cases and 6 population controls (Figure 1A), identifying 5001 CpG sites differentially methylated (adjusted p-value <0.05). As expected, the CLL methylome was characterized by a widespread hypomethylation, but by restricting the analysis to the regulatory regions, a switch towards hypermethylation, especially in the CpG islands (CGIs), was evident (Figure 2). Since the genome-wide methylation analysis was conducted on whole blood samples, which are characterized by a high level of cellular heterogeneity, we were not able to detect CpG islands significantly (adjusted p-value <0.05) hypermethylated in CLL samples compared to normal control samples. For this reason, we selected the 100 top-ranked differentially methylated CGIs (Supplementary Table 1).

As the total number of lymphocytes is much higher in tumor samples than in control samples, it must be taken into account in the differential methylation analysis. For this reason, we repeated the differential methylation analysis using the absolute lymphocyte count data as a covariate in the limma model. This analysis identified 7886 CpG sites differentially methylated (adjusted p-value <0.05) between CLL and control samples. There was no substantial change in the list of the 100-top ranked differentially methylated CGIs (Supplementary Table 1) detected in this second analysis, which indicates that the differences in the DNA methylation pattern we observed between CLL cases and controls were not due to the different number of circulating lymphocytes.

### *SHANK1* methylation alteration as a potential tumor biomarker

In order to be able to subsequently carry out a functional study, and having little material available, we focused our attention on the most altered CGI, located in the gene body of *SHANK1* (chr19:51198143-51198460, referred to hg19 assembly). This CGI was hypermethylated in the CLL samples, and it showed the highest mean differential methylation value (Δβ = 0.29) between CLL and control samples (Table 1 and Supplementary Table 1).

**Table 1:** *SHANK1* differential methylation data in the datasets analyzed

Groups	Mean Δβ	p-value	adj p-value
18 CLLs *vs* 6 normal controls (experimental dataset)	0.29	0.0023	0.2641
139 CLLs *vs* 20 normal controls (validation dataset) [6]	0.26	8.66e-12	2.43e-10
59 U-CLL *vs* 6 CD5+NBC/NBC (validation dataset) [8]	0.38		FDR<0.05 [8]
82 CLL/SLL yr before diagnosis *vs* matched controls (experimental predictive dataset)	0.047	0.00863	0.0921
438 MBCN cases yr before diagnosis *vs* matched controls (experimental predictive dataset)	0.03	4.97e-07	7.47e-05

Adj: adjusted; CLL: chronic lymphocytic leukemia; NBC: naïve B cells; SLL: small lymphocytic lymphoma; yr: years; MBCN: mature B-cell neoplasms

**Figure 3:**
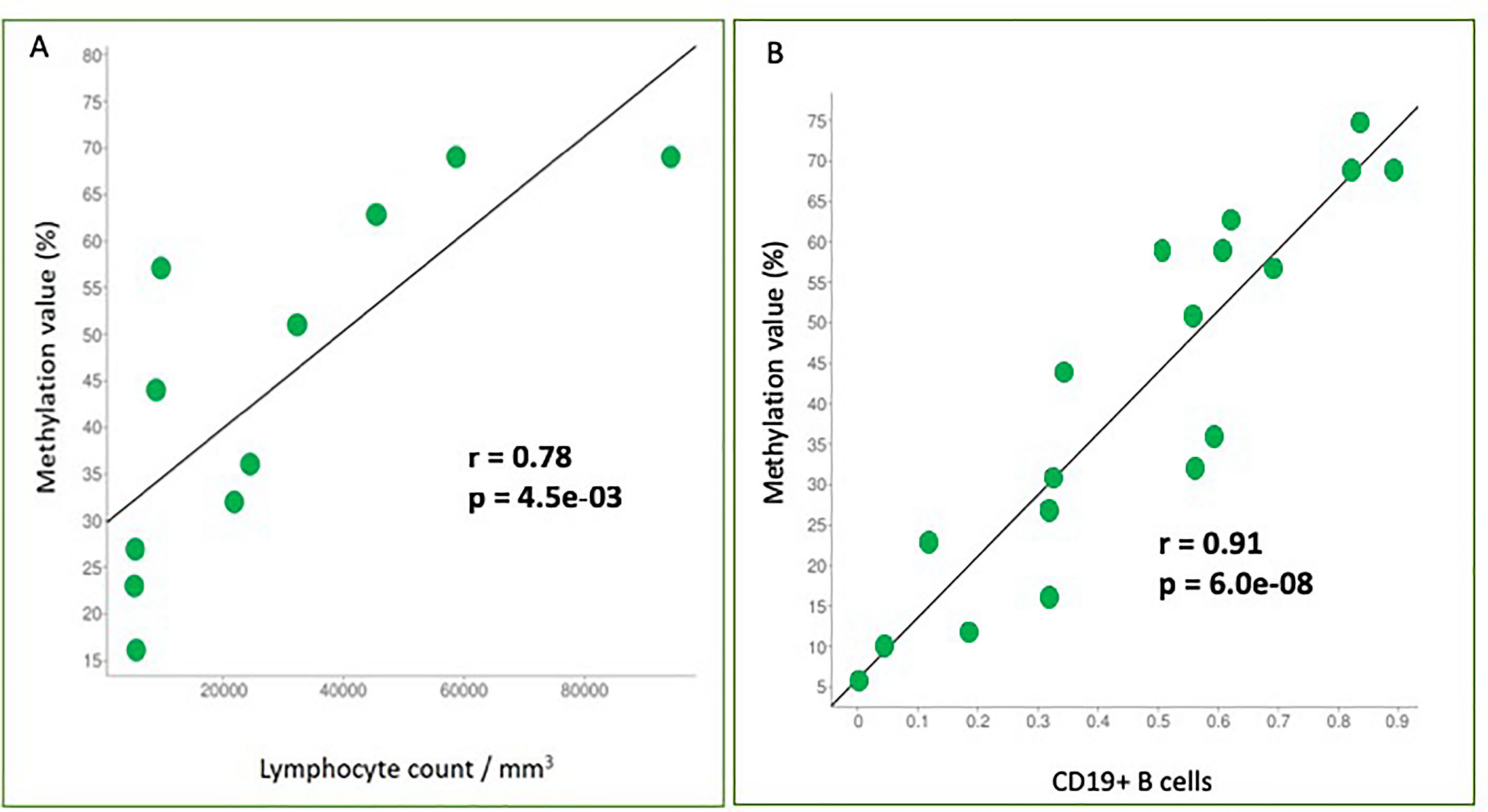
Correlation analyses between two variables. **(A)** Correlation analysis between methylation percentage and absolute lymphocyte count. *SHANK1* methylation values (%) are plotted against absolute lymphocyte count values. Pearson correlation coefficient (r) shows a significant positive correlation (r = 0.78, p-value = 0.0045); **(B)** Correlation analysis between methylation percentage and CD19+ B-cells. SHANK1 methylation values (%) are plotted against CD19+ cells contribution values. Pearson correlation coefficient (r) shows a significant positive correlation (r = 0.91, p-value = 6,00e-08).

We conducted a correlation analysis between the methylation percentage and the absolute lymphocyte count revealing a significant positive correlation (r = 0.78, p-value = 0.0045) (Figure 3A) between the two variables.

Since this methylation alteration was detected analyzing whole blood samples and a differential cell type content has an effect on DNA methylation profile, we investigated cell type heterogeneity in our samples. The relative contributions of each principal immune components of whole blood (B cells, granulocytes, monocytes, NK cells, and T cells subsets) in each sample were estimated by applying the algorithm of Houseman et al. [6] based on the distinctive methylation profiles of each cell type.

As expected, granulocytes, the most abundant leukocytes in peripheral blood in a healthy condition [7], contributed for most of cell type composition in normal blood control samples (data not shown).

On the other hand, tumor samples with a high lymphocyte count, showed a high CD19+ B-cells contribution and higher *SHANK1* methylation values, compared to samples with low CD19+ B-cells contribution (Table 2). Correlation analysis confirmed that there was a strong positive correlation between CD19+ B-cells contribution and *SHANK1* methylation values (r = 0.91, p-value = 6,00e-08) (Figure 3B).

**Table 2:** Comparison between estimated relative leucocyte contributions, lymphocyte count and *SHANK1* methylation values in CLL samples

Sample ID	CD14+ monocytes	CD19+ B cells	CD4+ T cells	CD56+ NK cells	CD8+ T cells	Granulocytes	Lymphocyte count/mm^3^	*SHANK1* Methylation value (%)
304012_002	0,104	0,609	-0,010	0,001	0,000	0,245	NA	59
304012_007	0,076	0,114	-0,001	0,011	0,000	0,797	5050	23
304012_030	0,061	0,556	0,000	0,040	0,000	0,355	32380	51
304012_048	0,074	0,693	0,000	0,049	0,000	0,122	9580	57
304032_083	0,084	0,837	0,038	0,000	0,000	0,002	NA	75
304012_088	0,062	0,621	-0,008	0,050	0,000	0,289	45330	63
304012_092	0,099	0,562	0,000	0,146	0,000	0,154	21830	32
304032_100	0,061	0,506	0,000	0,150	0,000	0,323	NA	59
304032_104	0,019	0,323	0,002	0,323	0,000	0,313	NA	31
304012_112	0,073	0,314	0,000	0,056	0,000	0,627	5270	27
304012_114	0,057	0,594	0,000	0,092	-0,001	0,291	24360	36
304032_132	0,000	0,000	-0,016	0,000	0,000	0,988	NA	6
304032_134	0,070	0,185	0,000	0,049	0,000	0,720	NA	12
304012_188	0,089	0,318	0,000	0,104	0,000	0,484	5410	16
304012_193	0,047	0,890	-0,028	0,000	-0,006	0,015	58880	69
304012_196	0,090	0,342	0,000	0,099	0,000	0,549	9060	44
304012_198	0,065	0,042	0,096	0,157	0,000	0,672	NA	10
304012_475	0,068	0,821	0,000	0,001	0,000	0,000	94100	69

Individual characteristics such as smoking status, age and BMI can affect DNA methylation and should be used for adjustment in differential methylation analysis. Since our sample size was small, we did not perform an adjustment of *SHANK1* methylation values using these data. However, it can be observed that differences in *SHANK1* methylation values in cases and between cases and controls are not associated to differences in smoking status, age or BMI (Table 3).

**Table 3:** Clinical and immunophenotypic characteristics of the samples used for methylome analysis

	Clinical and immunophenotypic characteristics											
**CLL patients**	**Sample ID**	**Age**	**Sex**	**Smoking status**	**BMI**	**CD5+ (%)**	**CD5+/CD19+ (%)**	**CD23+** **(%)**	**CD38+ (%)**	***IGHV* mutational ** **status**	**Lymphocyte count/mm^3^**	***SHANK1*** ** Methylation value (%)**
	304012 002	67	M	No Smoker	19,6	NA	NA	NA	NA	NA	NA	59
	304012 007	81	M	No Smoker	23,8	93.30	71.50	72.10	42	Positive	5050	23
	304012 030	67	M	No Smoker	27,2	98.60	92	92	2.6	Negative	32380	51
	304012 048	64	F	No Smoker	26,7	94.1	78.70	75.40	15	Negative	9580	57
	304032 083	75	M	Smoker	32,9	NA	NA	NA	NA	NA	NA	75
	304012 088	79	M	No Smoker	33,8	98.2	91	91.4	4.7	Negative	45330	63
	304012 092	64	M	No Smoker	26,9	88.7	64.8	63.7	10	Positive	21830	32
	304012 112	83	F	No Smoker	NA	34	6	70.3	14	Negative	5270	27
	304012 114	73	M	No Smoker	25,5	Negative/ weak	NA	65	16	Negative	24360	36
	304012 188	43	F	No Smoker	21,0	97	87	88	9	Positive	5410	16
	304012 193	52	M	Smoker	30,8	56	NA	NA	NA	Negative	58880	69
	304012 196	58	F	No Smoker	18,6	68	54	76	NA	Negative	9060	44
	304012 198	48	F	No Smoker	21,9	NA	NA	NA	NA	NA	NA	10
	304012 475*	60	M	No Smoker	23,0	97.9	94.60	Partially expressed	Weak	Positive	94100	69
**Controls**	304012 357	37	F	No Smoker	24,7	–	–	–	–	–	–	9
	304012 368	68	M	No Smoker	30,8	–	–	–	–	–	–	10
	304012 427	47	M	No Smoker	26,8	–	–	–	–	–	–	10
	304012 429	69	M	No Smoker	28,1	–	–	–	–	–	–	13
	304012 448	67	F	No Smoker	21,7	–	–	–	–	–	–	16
	304012 455	20	M	Smoker	21,7	–	–	–	–	–	–	8

For 4/18 patients with CLL diagnosis used for methylome analysis, clinical and immunophenotypic characteristics were not available. * this patient was firstly diagnosed as Follicular lymphoma.

To validate and increase the robustness of our data, we analyzed the methylation data for this CGI, obtained by Kulis et al. [8], of 139 CLLs (≥95% neoplastic cells) and 20 non-tumoral samples (normal B-cells from peripheral blood including total B cells and various subtypes of B-cells) (Figure 1B). Methylation data were retrieved from the ICGC Data Portal (https://dcc.icgc.org), DCC Data Release 27, DCC Project Code: CLLE-ES. We detected a Δβ value of 0.26 (p-value = 8.66e-12, adjusted p-value = 2.43e-10), confirming *SHANK1* hypermethylation in CLL (Table 1).

In addition, Kulis et al. identified the same CGI as hypermethylated in a subgroup of 59 CLL with a low or absent *IGHV* mutational load (U-CLLs) compared to 6 naïve B cells (CD5+NBC/NBC) (false discovery rate <0.05) [8] (Figure 1B, Table 1).

**Figure 4:**
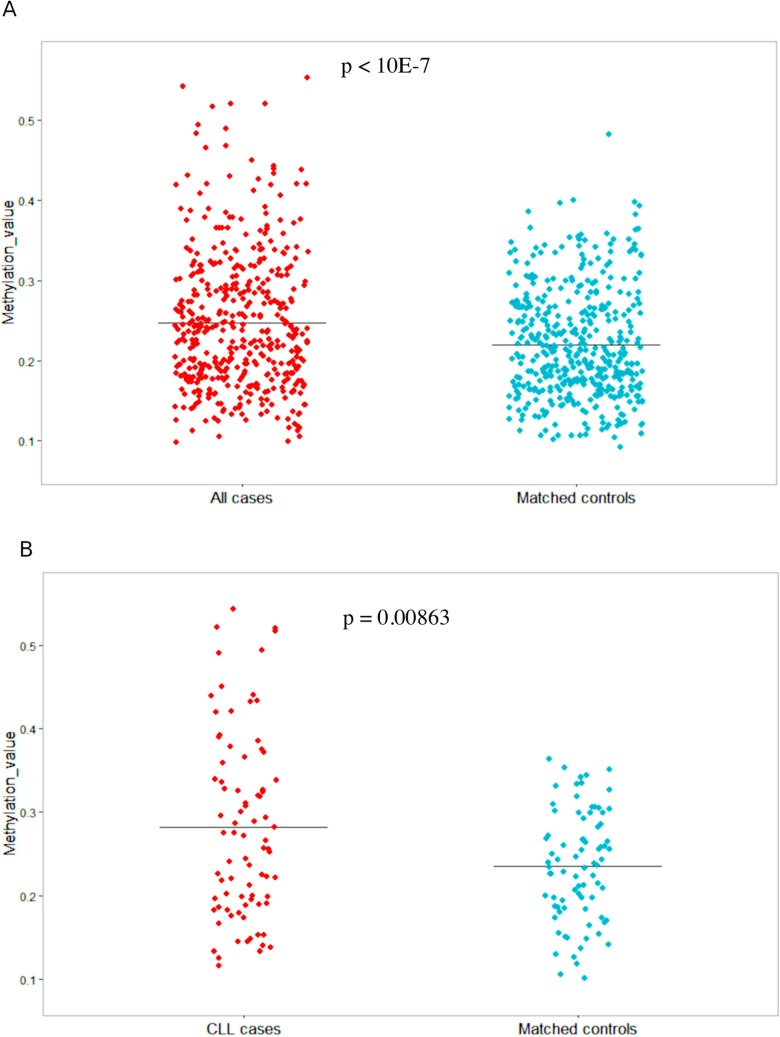
*SHANK1* methylation values in 438 MBCN cases/controls and the 82 CLL/SLL cases and controls. **(A)** Jitter plot showing methylation values (Y-axis) in all cases and their matched controls; **(B)** Jitter plot showing methylation values (Y-axis) in CLL cases and their matched controls.

To evaluate the potential role of *SHANK1*-associated CGI as a potential predictive biomarker, we conducted a differential methylation analysis between cases and matched controls in blood collected at baseline entry into the prospective cohort study (Figure 1A), finding a gain of methylation in the *SHANK1* CGI (chr19:51198143-51198460) detectable in blood samples collected years before diagnosis of 82 CLL and small lymphocytic lymphoma (SLL) (Δβ = 0.047, p = 0.00863, adjusted p-value = 0,0921) (Table 1). Moreover, by extending the methylation analysis to the larger series of 438 mature B-cell neoplasms (MBCN) cases, including the 82 CLL/SLL, we confirmed significant differential methylation of the same CGI (Δβ = 0.03, p-value < 10^-7^, adjusted p-value = 7.47e-05) between cases and controls (Table 1). Figure 4 shows the methylation values observed in the extended cohort (Figure 4A) and in the CLL/SLL subgroup (Figure 4B).

### *SHANK1* gene expression analysis

**Figure 5:**
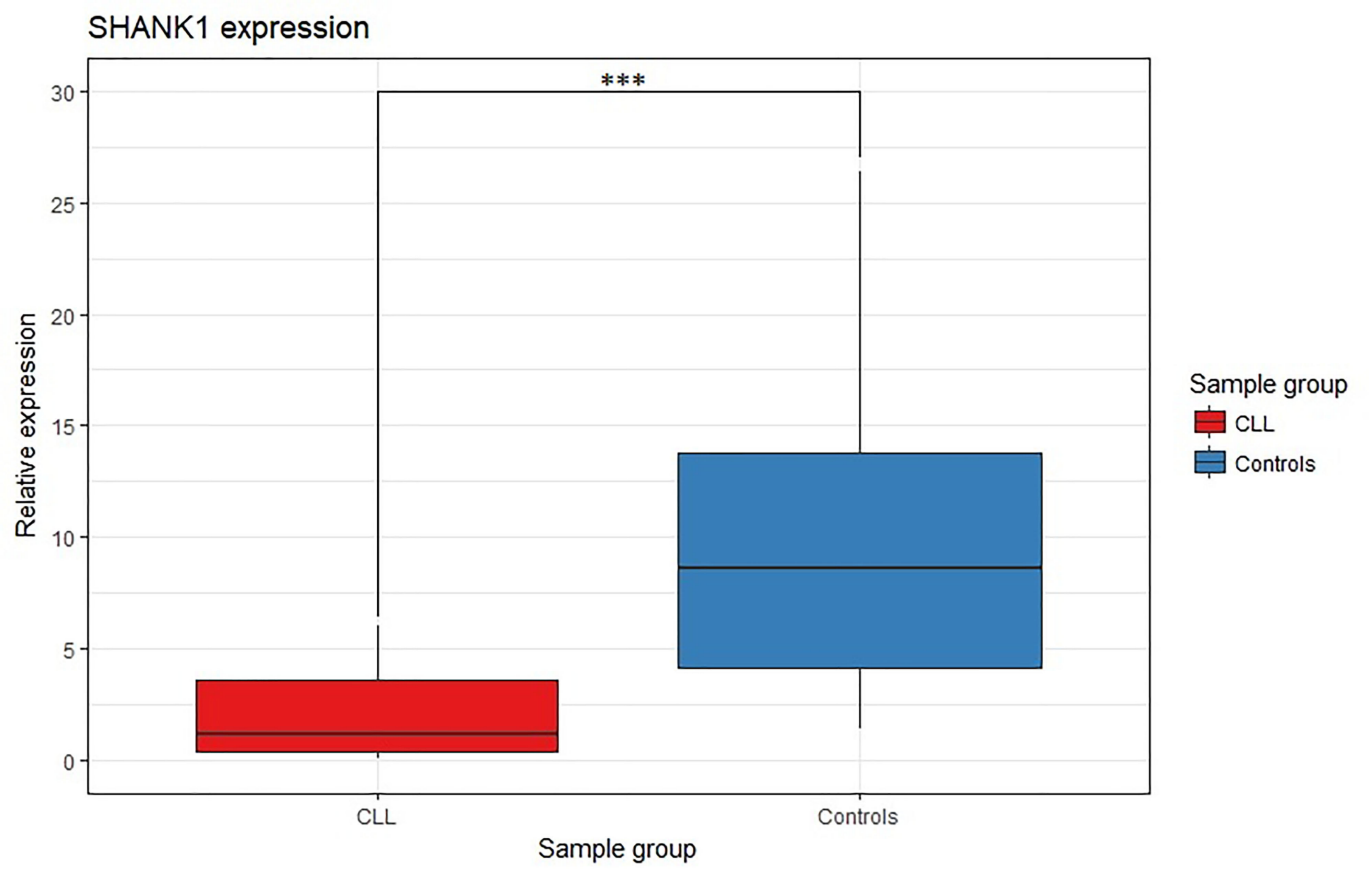
*SHANK1* differential gene expression analysis between CLL and control samples. Box plot *SHANK1* fold change values for CLL and controls samples. *** indicates p-value < 0.0001.

To investigate the impact of *SHANK1*-associated CGI hypermethylation on gene expression, we tested the gene expression level of *SHANK1* in 27 CLL cases and 16 non-tumor subjects by qRT-PCR (Figure 1A). *SHANK1* showed a significant almost 8-fold down-regulation (p-value <0.0001) in CLL cases compared to controls (Figure 5).

*SHANK1* downregulation was also confirmed using *GUSB* as reference gene in a subgroup of samples (data not shown).

To validate *SHANK1* downregulation observed in our CLL samples, we analyzed RNA-seq data publicly available. Differential gene expression data of ten CLL specimens versus five normal peripheral blood CD19+ B cells were retrieved from GEO database under the accession number GSE70830. In this dataset, *SHANK1* gene showed a non-significant differential expression between CLL and control samples.

Gene expression data of a large dataset [9] including 98 CLL and three subtypes of normal B cells (naïve, memory IgM/IgD, and memory IgG/IgA), from three different healthy individuals revealed that only three CLL samples show more than 10 reads and most CLL and normal samples have zero reads, therefore making these data unsuitable for a validation of *SHANK1* downregulation.

Whole blood gene expression data from The Genotype-Tissue Expression (GTEx) Portal (https://gtexportal.org) revealed that *SHANK1* is weakly expressed in whole blood (median Transcripts Per Kilobase Million (TPM): 0.030, number of samples: 407). RT-qPCR represents the most precise and sensitive method to detect differences in gene expression for weakly expressed genes. On the other hand, the accurate quantification of gene expression by RNA seq depends on the sequencing depth and it has been suggested that sequencing up to 100 million reads can be necessary to quantify precisely genes and transcripts that have low expression levels [10].

## DISCUSSION

Aberrant methylation patterns, one of the most striking features of cancer, might represent useful biomarkers for prediction of cancer risk, early diagnosis, prognosis, and for prediction of response to treatment and cancer relapse. Furthermore, as DNA methylation is an early epigenetic reversible modification, specific drugs can be developed to restore the DNA methylation pattern of normal cells at the initial stages of carcinogenesis.

A substantial body of evidence suggests methylation changes as innovative biomarkers for both early cancer diagnosis and prognosis [11]. Some such changes have already shown clinical relevance, such as *BRCA1* in breast cancer, *MGMT* in glioblastoma multiform, and *SEPT9*, which has been approved by the Food and Drug Administration (FDA) for the diagnosis of colon cancer. Since global DNA methylation is similar in resting and proliferative compartments [12], aberrant methylation patterns may be an early event in CLL, as suggested by its occurrence in blood samples collected years before diagnosis of mature B-cells neoplasm [3] and CLL [4].

In addition, methylation alterations in CLL, such as *ZAP70* [13] and *HOXA4* [14], have been proposed as prognostic biomarker, and differential methylation profiles are used to classify CLL patients in three molecular CLL subtypes having different clinical features and deriving from B-cell subpopulations at different stages of differentiation [5,15,16]. To our knowledge, biomarkers for detecting CLL at an early stage, which would permit the initiation of therapy in the first phases of the disease, are still lacking. In our study, we identified several CGIs differentially methylated (Supplementary Table 1), and we confirmed a genome-wide hypomethylation in CLL (Figure 2), as previously observed in other studies [8].

Although three CGIs resulted significantly hypomethylated in CLL samples (Supplementary Table 1), we do not suggest these alterations as diagnostic biomarkers since the difficulty of setting cut-off values to consider a sample as hypomethylated, especially when we want to look for an alteration that correlates, as in our case, with the number of tumor cells, then a biomarker that increases with the increase of tumor cells number. On the other hand, hypermethylated CGI showing high methylation values in tumoral samples could be more easily employed as tumoral biomarker.

Our most striking finding was the hypermethylation of a CGI located in the gene body of *SHANK1* in CLL samples. Although this differential methylation was not statistically significant after correction for multiple testing probably due to the fact that the sample size in our study was small, we were able to replicate our finding by *in silico* analysis of a larger CLL series [8]. This type of approach has proved to be successful in the identification of other highly specific and sensitive tumor biomarkers [17–20]. Moreover, the robustness of our data was verified in different validation and exploratory sets (Table 1). For these reasons, it is important to mention that, although we are aware of the importance of significance threshold for epigenome-wide studies (EWAS) [21], the methylation alterations detected are somatic epimutations, and even at the onset of the disease, thus the heterogeneity of the tumor, and in particular, as in this case, a blood tumor, needs to be taken into account. In our previous work [19], we have shown that in a heterogeneous tissue, such as adenoma, the methylation alterations detected were not statistically significant after multiple testing adjustment. In contrast, the same methylation alteration detected in colorectal carcinomas resist to any correction for multiple testing. In fact, adenomas include a mixture of cells showing methylation alterations and cells not showing the alterations compared to colorectal carcinomas where many tumor cells present the epimutations.

*In silico* replication of our result analyzing data including CLL samples with ≥95% neoplastic cells and control normal B cells [8], confirmed that the detected *SHANK1* methylation alteration belongs to neoplastic cells. The fact that we were able to detect this methylation alteration in CLL whole blood samples supported its potential use as a diagnostic biomarker to be introduced in clinical practice without the need of performing an expensive method such as cell sorting. Interestingly, Kulis et al [8] have shown that, in a differential methylation analysis between U-CLL samples and naïve B cells of control samples, the same *SHANK1* CGI, identified as altered in our study, was significantly hypermethylated in U-CLL. *IGHV* mutational status is a prognostic biomarker in CLL: while patients with a high level of *IGHV* mutation (called as M-CLL) have a favorable prognosis, U-CLL is usually associated with poor outcomes. In our case series, patients with *IGHV* mutations had an average *SHANK1* CGI methylation value of 35.0%, while it was 49.6 % in *IGHV* non-mutated patients (Table 3). Also, removing an outlier (69.0% methylation), probably due to the fact that this patient was first diagnosed with Follicular lymphoma, resulted in the methylation average dropping to 23.7%. Thus, *SHANK1* methylation might be correlated with clinical outcomes in CLL. In this regard, it is interesting to note that we also observed that the β value for the altered CGI in *SHANK1* correlates positively with the peripheral lymphocyte count at diagnosis (Figure 3), which also prompted us to repeat the differential methylation analysis by including the lymphocyte count as a covariate; this second analysis confirmed the same CGIs as the most affected by methylation changes (Supplementary Table 1). Inference of cell type contributions on tumor whole blood samples revealed that there was a strong positive correlation between CD19+ B-cells contributions and *SHANK1* methylation values (Table 2). It is estimated that the increased number of lymphocytes, that is observed in the lymphocyte count at diagnosis, is mainly due (> 80%) to the proliferation of neoplastic cells. In fact, in an analysis of 110 patients with an absolute lymphocyte count of at least 5 x 10^9^/L, Shanafelt et al. have shown that monoclonal B-cells were more than 86% of B-cells, while polyclonal B-cells represented only a small fraction of total B-cells [22]. Therefore, *SHANK1* methylation might be a useful molecular biomarker in the personalized quantification of tumor aggression in CLL.

Aberrant methylation of genes implicated in MBCN is detectable in blood samples collected many years before diagnosis [3, 4]. We found that within a prospective cohort, there was a significant gain of methylation (differential methylation of 3%) in the same *SHANK1* CGI detectable in peripheral blood collected many years prior to diagnosis with MBCN (Figure 4A). In a subgroup of CLL/SLL cases within the prospective MBCN cohort, there was an even more pronounced gain of methylation (differential methylation of 4.7%) compared with matched unaffected controls (Figure 4B).

Since there is an overlap between *SHANK1* methylation levels in cases and controls, *SHANK1* cannot be suggested as a predictive biomarker at individual levels. However, 210/438 (48%) MBCN samples showed a differential methylation greater than the average Δβ (0.03), ranging from 0.031 to 0.36, and 38/82 (46%) CLL/SLL samples showed a differential methylation greater than the average Δβ (0.047), ranging from 0.06 to 0.33. It is important to mention that each case is matched to one control. Since we found that *SHANK1* methylation values are positively correlated to lymphocyte counts at diagnosis and patients with poor prognosis according to *IGHV* mutational status showed higher methylation values, we can speculate that *SHANK1* might be able to predict aggressive forms of disease years before diagnosis. Further studies including *SHANK1* methylation status before diagnosis, at diagnosis and follow-up data are needed to investigate this hypothesis.

This difference of methylation is in line with, and even greater than that observed in other genes years before cancer diagnosis [3]. Thus, *SHANK1* might be a potential predictive biomarker of CLL risk. Although *SHANK1* methylation data at diagnosis in other MBCNs were not available in our study, we can speculate that methylation of this gene is an early event in leukemogenesis.

The use of an epigenetic biomarker proving to be so traceable and informative without cells sorting, is particularly useful for preventive purposes. Extensive screening aimed to identify individuals at risk for CLL would not make much sense using detectors requiring cells sorting, while it is possible to find alterations in the methylation pattern of this specific CGI already years before the onset, starting from whole blood.

*SHANK1* is one of the three members of the SHANK (SH3 And Multiple Ankyrin Repeat Domains) gene family. Their respective proteins, SHANK1, SHANK2 and SHANK3 act as scaffold proteins and have a fundamental role in the formation, development and function of neuronal synapses. Mutations of the *SHANK* gene family are associated to developmental disorders, such as autism and schizophrenia. Investigating the association between *SHANK* genes methylation and their expression, Beri et al [23] showed that, although all these genes present several methylated CpG sites, only *SHANK3* was highly methylated in tissues where its expression was low or absent, suggesting that methylation might regulate tissue-specific *SHANK3* expression.

In our work, the gene expression analysis revealed that *SHANK1* was significantly down-regulated in CLL. The inability to validate this data, *in silico*, may be due to the fact that a gene already normally repressed, if further down-regulated, is hardly detectable by means of whole exome / whole transcriptome NGS / microarrays techniques. It will therefore be important to verify our observation on a larger database, by means of a targeted expression study and an ultrasensitive technique such as ddPCR. The association between methylation and gene expression is very complex and, while hypermethylation of CGIs located in gene promoters is usually associated with gene down-regulation, hypermethylation of intragenic CGIs has been correlated either positively and negatively with gene expression [8, 24–27]. Intragenic DNA methylation might have a role in several molecular processes, such as regulation of cell-context specific alternative promoter in gene bodies [28], expression of intragenic non-coding RNA [29–32] and transposable elements [33], alternative splicing [34], alternative polyadenylation sites [35], and enhancer activation [36, 37]. It has also been shown that intragenic nucleosomes with H3K36 trimethylation (H3K36me3), a histone modification associated with transcript elongation, recruit DNA methyltransferases [38], suggesting that DNA methylation is unable to block transcript elongation. In CLL, Kulis et al [8], have shown that, in absence of CGI-promoter methylation, methylation of CpGs located in the gene bodies of around 900 genes shows a significant correlation (either positive or negative) with gene expression.

**Figure 6:**
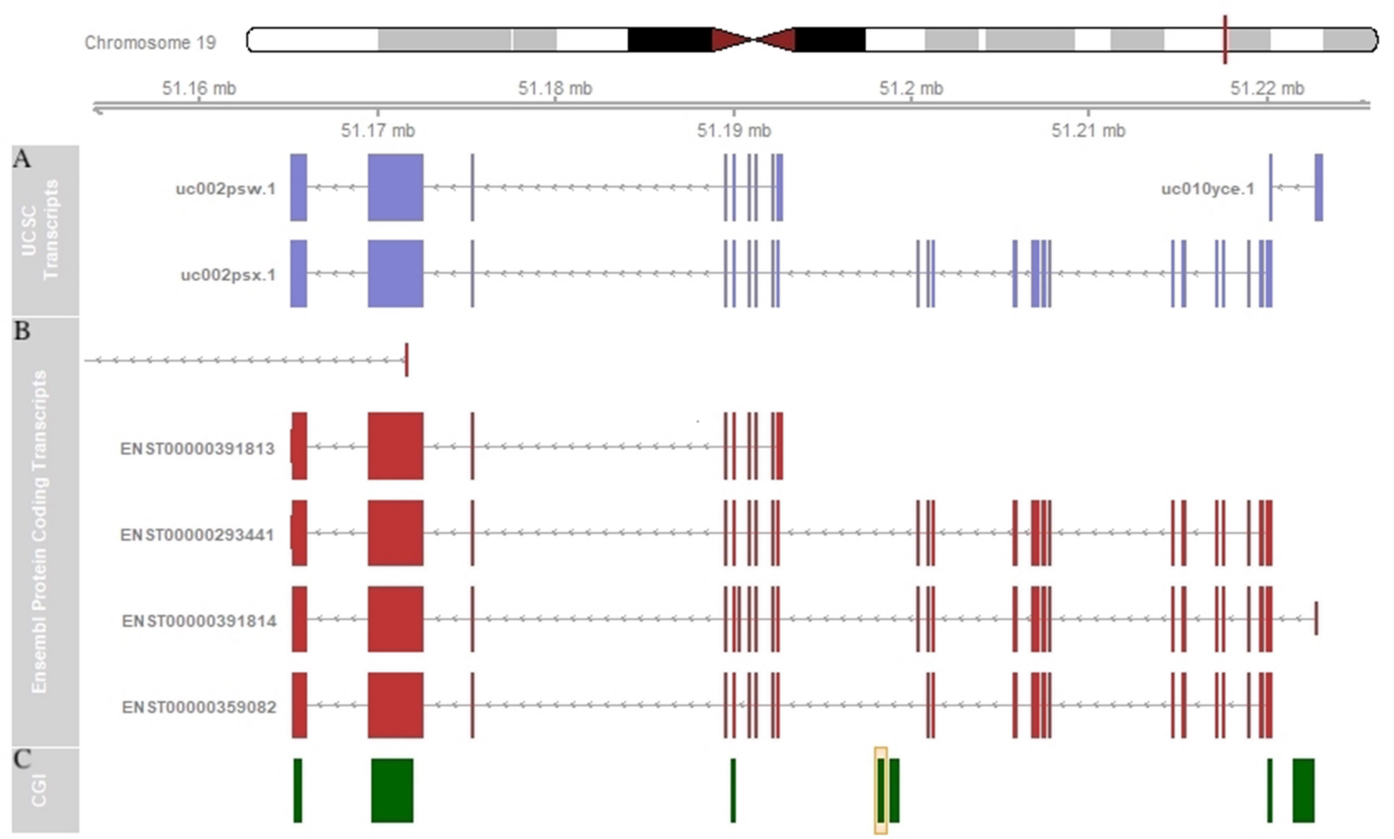
*SHANK1* schematic representation. **(A)** *SHANK1* transcripts representation in UCSC Genome Browser. **(B)** *SHANK1* protein coding transcripts representation in Ensembl Genome Browser. **(C)** *SHANK1*-associated CGI annotation in UCSC Genome Browser. The orange box indicates the CGI found altered in our study.

Since methylation of intragenic CGIs might be inversely correlated with the expression of an alternative transcript and positively regulated with the expression of the main transcript [39], it would be interesting to investigate the association between the methylation of the intragenic CGI of *SHANK1* and the expression of alternative transcripts. *SHANK1*, like the other two *SHANK* genes, *SHANK2* and *SHANK3*, presents a complex transcriptional structure. This gene contains two different promoters, which generate the longest protein isoform (called *SHANK1A*) and the shortest one (called *SHANK1B*), and several splicing sites generating alterative transcripts (Figure 6). *SHANK1* is mainly expressed in the brain (https://www.gtexportal.org/home/gene/SHANK1). The CGI found to be hypermethylated in our CLL cases is located in the gene body of the main transcript (uc002psx.1 or ENST00000293441), but it is upstream of the shorter transcript (uc002psw.1 or ENST00000391813) (Figure 6). Thus, we speculate that the hypermethylation of the intragenic CGI might be associated with a down-regulation of this shorter transcript. Clearly, a gene expression study to accurately quantify each *SHANK1* alternative transcript in a large number of CLL samples and controls is warranted to elucidate the impact of this intragenic CGI hypermethylation in gene expression regulation.

To date, it is difficult to hypothesize whether the identified methylation alterations are really associated with a different gene expression profile and what this may possibly influence from a functional point of view. The putative activation or deactivation of isoforms that do not respect the normal cell differentiation program could obviously affect the cellular functionality, so as to induce it to a neoplastic iter. What is certain is that at the moment these alterations can provide useful biomarkers for the early diagnosis of a neoplasia, as well as, in the case of the marker we identified, associate with a different prognosis and even giving the possibility of predicting a risk of disease onset.

Other *SHANK1*-associated CGIs are reportedly differentially methylated in other cancers, and they have been proposed as tumor biomarkers [19, 40]. The most plausible scenario would be that methylation changes in different *SHANK1*-associated CGIs would be related to different types of cancer, thus serving as specific signatures for different types of tumors.

In summary, our results suggest *SHANK1* as a promising tumor biomarker for CLL early diagnosis. Since no screening tests are recommended for CLL and patients can be asymptomatic, it is difficult to diagnose CLL in the first phases of the disease. The introduction of a methylation-based diagnostic biomarker in clinical practice could allow an early detection of CLL cases and the initiation of adequate therapeutic treatments in the first phases of the disease. *SHANK1* hypermethylation can be easily found analyzing whole blood samples without cell sorting, supporting its utility in clinical practice. Moreover, since *SHANK1* methylation levels correlated with lymphocyte count at diagnosis it can be particularly useful in the recognition of the most aggressive forms of the disease showing a fast progression. Finally, the detection of *SHANK1*-associated CGI gain of methylation many years before diagnosis suggests that *SHANK1* methylation levels may be predictive of CLL development. Thus, a screening test based on *SHANK1* methylation levels assessment could be developed to monitor people at risk, such as people over age 50, or subjects presenting familiarity for the disease. Further studies on sequential samples before diagnosis, at diagnosis and during disease course are needed to monitor *SHANK1* methylation levels and ascertain its involvement in CLL progression.

## MATERIALS AND METHODS

### Tumor specimens

Eighteen Italian patients (10 men and 8 women, mean age at diagnosis: 65.3±12.3) with a diagnosis of CLL donated a blood sample during their first visit at the outpatient ambulatory of the Hematology department of the A. Businco Oncology Hospital, Cagliari (Sardinia, Italy). All patients were diagnosed using the 2008 WHO Classification of lymphoid neoplasms criteria. [41]

Blood samples from six controls (mean age: 51.3±20.2), selected from the 151 participating in a population-based case-control study in the same area of CLL patients, were included in the genome-wide methylation analysis.

Demographic characteristics (age, sex, smoking status and BMI) of CLL patients and controls and clinical and immunophenotypic characteristics of CLL samples are reported in Table 3.

Mean age (p-value = 0,0711; Student t test), sex (p-value = 1,0000; Fisher’s exact test), smoking status (p-value = 1,0000; Fisher’s exact test) and mean BMI (p-value = 0,9613; Student t test) were not statistically significant different between cases and controls.

In addition, blood samples were available for 27 out of the 29 CLL incident cases (mean age at diagnosis: 65.1±9.3) recruited for the same study at participating Italian hospitals in Novara, Florence, Perugia and Cagliari, and for a random sample of 16 out of the 455 population controls from the same areas, who accepted taking part to the full study protocol. We conducted the gene expression analysis for these 27 CLL cases and 16 controls.

We also tested the hypothesis that the selected *SHANK1* CpG island (CGI) might undergo methylation changes prior to the clinical manifestation of the disease using a case control study nested within a prospective cohort study of 438 samples of incident mature B-cell neoplasms (MBCN), including 82 CLL and small lymphocytic lymphoma (SLL) cases, considered two forms of the same disease only differing by the location where the cancer primarily occurs, and matched (individually matched to cases at 1:1 ratio based on age at enrollment, gender, ethnicity and DNA source) controls. Additional information about cases and controls demographic characteristics and matching criteria can be found in Woong Doo et al. [3]. In this cohort, the mean time between blood collection and diagnosis was 9.5 years (range 0.6-17.8 years) for CLL cases and 10.6 years (range 0.2-20 years) for the entire MBCN cohort. DNA was collected predominantly from whole blood samples and analyzed as previously described. In particular, of the 976 samples, 632 were processed from whole blood, 234 from Ficoll-separated mononuclear cells and 10 from buffy coat. [3]

All the biological samples analyzed were obtained with written informed consent signed by patients/study participants and ethical approval granted by the “Comitato Etico Azienda Ospedaliero Universitaria di Cagliari” (269/09/CE, 26/05/2009).

### DNA and RNA extraction

DNA was isolated from peripheral whole blood lymphocytes using the DNA extraction 500 arrow® Kit (DiaSorin Ireland Ltd) kit. DNA was quantified with NanoDrop (NanoDrop Products Thermo Scientific Wilmington, DE) and by fluorometric reading (Quant-iT™ PicoGreen® dsDNA Assay Kit).

RNA was extracted from PBMCs (Mononuclear cell fractions, isolated over a Ficoll-Hypaque density gradient) using Qiagen RNAeasy mini kit (Qiagen, Hilden, Germany) and quantified using NanoPhotometer (NanoPhotometer™Pearl, Denville®, Denville Scientific, Holliston, MA).

### DNA methylation analysis

The DNA extracted was bisulfite converted using the EZ DNA Methylation gold Kit (Zymo Research, Irvine, CA, USA) according to manufacturer’s instructions. Bisulfite converted DNA was hybridized to Illumina Infinium HumanMethylation450 BeadChips (450K), following the Illumina Infinium HD Methylation protocol. Hybridization fluorescent data were read on an Illumina HiScan SQ scanner.

Illumina Methylation 450K raw data were analyzed using the RnBeads analysis software package as previously described. [42, 43] Methylation levels [beta values (β)] were estimated as the ratio of signal intensity of the methylated alleles to the sum of methylated and unmethylated intensity signals of the alleles. The β values ranges from 0 (no methylation) to 1 (100% methylation).

Differential methylation analysis was conducted on the CpG sites and other 4 genomic regions (tailing, genes, promoters and CpG Islands, so called CGIs). CpG-level p-values were corrected for multiple testing using the false discovery rate (FDR) method. CpG-specific uncorrected p-values within a given genomic region were combined to obtain aggregate p-values for each genomic region and then corrected for multiple testing. [42, 43] Based on the absolute and relative effect size of the differences between the study groups, RnBeads combines statistical testing with a priority ranking scheme to assign a combined rank score for differential DNA methylation to each analyzed CpG site and genomic region. [42] The generated priority-ranked list was used to select the top-100 ranked differentially methylated CGIs. The selected CGIs were annotated to nearest genes by using R annotation package FDb.InfiniumMethylation.hg19. [44]

An additional methylome analysis was performed adding absolute lymphocyte count data as a covariate in the limma analysis of differential DNA methylation. [42, 45] DNA methylation analysis for the samples in the nested case-control study were also performed on the Illumina HM450K Infinium array as previously described. [3]

Inference of cell type contributions was conducted using RnBeads applying the method of Houseman et al. [6]. This method estimated the cell type contributions of whole blood samples based on methylation profiles of a sorted blood cell types reference. [46] The validity of this method for estimating whole blood cells composition in CLL samples has been recently demonstrated. [4, 47]

### qRT-PCR

1μg RNA/sample was retro-transcribed using the High Capacity Kit (Applied Biosystems, Carlsbad, CA, USA). Gene expression analysis was performed by qRT-PCR, conducted on a DNA Engine Opticon 2 Real-Time Cycler (Bio-Rad, Hercules, CA, USA), using iQ™ SYBR® Green Supermix (Bio-Rad, Hercules, CA, USA) for each gene tested and for the reference genes. Actin Beta (*ACTB*) gene, which is one of the most often used reference genes in B-CLL gene expression studies [48, 49], was used as reference gene. A subgroup of tumoral and non-tumoral samples was also analyzed using Glucuronidase Beta (*GUSB*) as reference gene.

Primers used in this study to conduct qRT-PCR are the following:

- 5’-AGACCATCAGTGCAAGCGAA-3’ (*SHANK1* forward)

- 5’-GGGATCGAAGCTCGACTCAG-3’ (*SHANK1* reverse)

- 5’-AAATCTGGCACCACACCTTC-3’ (*ACTB* forward)

- 5’-AGCACAGCCTGGATAGCAAC-3’ (ACTB reverse)

- 5’-CACCTAGAATCTGCTGGCTACT-3’ (*GUSB* forward)

- 5’-AGAGTTGCTCACAAAGGTCACA-3’ (*GUSB* reverse)

Gene expression data were analyzed using the ΔΔCT method. We used a t-test for independent series to compare the average ΔCT of CLL cases and controls.

### Power calculation

The power was estimated using a two-sample t test power calculation. A dataset of 18 samples, those available to us as discovery set, would guarantee a statistical power of 0.8 to detect a differential methylation level of at least 25%, using a type I error of 10e-8 (which takes into account the need to correct for multiple test).

## SUPPLEMENTARY MATERIALS TABLE




