# Supplementary material for: Methylation alteration of SHANK1 as a predictive, diagnostic and prognostic biomarker for chronic lymphocytic leukemia
Source: Oncotarget. 2019 Aug 13;10(48):4987–5002. doi: 10.18632/oncotarget.27080 (PMC6697638; doi:10.18632/oncotarget.27080)
Supplement: Supplementary file 1 [file oncotarget-10-4987-s001.pdf]

Methylation alteration of *SHANK1* as a predictive, diagnostic and prognostic biomarker for chronic lymphocytic leukemia

SUPPLEMENTARY MATERIALS

Supplementary Table 1: 100-top ranked differentially methylated CpG islands in CLL discovery set

See Supplementary Table 1
