# Supplementary material for: Methylation alteration of SHANK1 as a predictive, diagnostic and prognostic biomarker for chronic lymphocytic leukemia
Source: Oncotarget. 2019 Aug 13;10(48):4987–5002. doi: 10.18632/oncotarget.27080 (PMC6697638; doi:10.18632/oncotarget.27080)
Supplement: Supplementary file 2 [file oncotarget-10-4987-s002.docx]

**Supplementary Table 1:** 100-top ranked differentially methylated CpG islands in CLL discovery set

| **DIFFERENTIAL METHYLATION ANALYSIS BEFORE COVARIATE ADJUSTMENT** | | | | | | | | **DIFFERENTIAL METHYLATION ANALYSIS AFTER COVARIATE ADJUSTMENT** | | | | | | | |
| --- | --- | --- | --- | --- | --- | --- | --- | --- | --- | --- | --- | --- | --- | --- | --- |
| **CpG Islands** | **Mean β CLL** | **Mean**  **β CTRL** | **Mean**  **Δβ** | **Comb. P-value** | **Comb. P-value adj.** | **Distance** | **Gene Symbol** | **CpG Islands** | **Mean β CLL** | **Mean**  **β CTRL** | **Mean**  **Δβ** | **Comb. P-value** | **Comb. P-value adj.** | **Distance** | **Gene Symbol** |
| chr19:51198144-51198460 | 0.41 | 0.11 | 0.30 | 0.0013 | 0.2518 | 0 | SHANK1 | chr19:51198144-51198460 | 0.41 | 0.12 | 0.29 | 0.0023 | 0.2641 | 0 | SHANK1 |
| chr9:116860474-116860695 | 0.35 | 0.06 | 0.29 | 0.0016 | 0.2746 | 0 | KIF12 | chr9:116860474-116860695 | 0.35 | 0.06 | 0.29 | 0.0024 | 0.2641 | 0 | KIF12 |
| chr4:5894072-5895116 | 0.45 | 0.16 | 0.29 | 0.0016 | 0.2746 | 0 | CRMP1 | chr4:5894072-5895116 | 0.43 | 0.15 | 0.28 | 0.0025 | 0.2671 | 0 | CRMP1 |
| chr2:200524002-200524204 | 0.38 | 0.10 | 0.28 | 0.0012 | 0.2518 | 101054 | FTCDNL1 | chr2:200524002-200524204 | 0.35 | 0.09 | 0.25 | 0.0014 | 0.2435 | 101054 | FTCDNL1 |
| chr12:127210779-127211651 | 0.43 | 0.15 | 0.27 | 0.0044 | 0.2933 | 3595 | LINC00944 | chr9:23850911-23851522 | 0.29 | 0.04 | 0.25 | 0.0018 | 0.2599 | 24847 | ELAVL2 |
| chr11:125774293-125774584 | 0.35 | 0.10 | 0.24 | 0.0017 | 0.2803 | 0 | DDX25 | chr11:125774293-125774584 | 0.33 | 0.09 | 0.24 | 0.0021 | 0.2641 | 0 | DDX25 |
| chr9:23850911-23851522 | 0.29 | 0.05 | 0.24 | 0.0019 | 0.2803 | 24847 | ELAVL2 | chr19:52452317-52452543 | 0.39 | 0.15 | 0.24 | 0.0056 | 0.2811 | 3305 | ZNF613 |
| chr20:13975769-13976287 | 0.34 | 0.10 | 0.24 | 0.0057 | 0.3057 | 0 | SEL1L2 | chr8:35092680-35093559 | 0.38 | 0.16 | 0.22 | 0.0052 | 0.2786 | 0 | UNC5D |
| chr19:52452317-52452543 | 0.39 | 0.15 | 0.23 | 0.0064 | 0.3057 | 3305 | ZNF613 | chr19:30865684-30866490 | 0.29 | 0.07 | 0.22 | 0.0012 | 0.2425 | 0 | ZNF536 |
| chr8:35092680-35093559 | 0.40 | 0.17 | 0.23 | 0.0030 | 0.2834 | 0 | UNC5D | chr8:120220389-120221222 | 0.32 | 0.10 | 0.22 | 0.0019 | 0.2605 | 0 | MAL2 |
| chr19:30865684-30866490 | 0.31 | 0.08 | 0.23 | 0.0015 | 0.2746 | 0 | ZNF536 | chr9:90589210-90589807 | 0.27 | 0.05 | 0.22 | 0.0001 | 0.1000 | 0 | CDK20 |
| chr8:120220389-120221222 | 0.33 | 0.11 | 0.23 | 0.0012 | 0.2518 | 0 | MAL2 | chr19:54485304-54486322 | 0.47 | 0.25 | 0.22 | 0.0015 | 0.2477 | 0 | CACNG8 |
| chr3:170303533-170303768 | 0.37 | 0.14 | 0.23 | 0.0063 | 0.3057 | 0 | SLC7A14 | chr8:24770909-24772547 | 0.30 | 0.09 | 0.22 | 0.0008 | 0.2062 | 0 | NEFM |
| chr9:90589210-90589807 | 0.29 | 0.06 | 0.23 | 0.0002 | 0.1151 | 0 | CDK20 | chr11:134201785-134202407 | 0.24 | 0.03 | 0.21 | 0.0006 | 0.1869 | 0 | GLB1L2 |
| chr8:24770909-24772547 | 0.32 | 0.10 | 0.23 | 0.0007 | 0.2184 | 0 | NEFM | chr13:24121204-24121465 | 0.34 | 0.13 | 0.21 | 0.0006 | 0.1777 | 23043 | TNFRSF19 |
| chr19:54485304-54486322 | 0.48 | 0.26 | 0.23 | 0.0011 | 0.2505 | 0 | CACNG8 | chr14:38724255-38725537 | 0.32 | 0.11 | 0.21 | 0.0031 | 0.2684 | 0 | CLEC14A |
| chr13:24121204-24121465 | 0.36 | 0.14 | 0.22 | 0.0009 | 0.2379 | 23043 | TNFRSF19 | chr5:16179065-16180420 | 0.30 | 0.09 | 0.21 | 0.0049 | 0.2754 | 0 | MARCH11 |
| chr14:38724255-38725537 | 0.34 | 0.12 | 0.22 | 0.0024 | 0.2803 | 0 | CLEC14A | chr6:1378446-1379318 | 0.24 | 0.04 | 0.20 | 0.0036 | 0.2706 | 10750 | FOXF2 |
| chr11:134201785-134202407 | 0.25 | 0.04 | 0.22 | 0.0007 | 0.2161 | 0 | GLB1L2 | chr5:174151479-174152364 | 0.32 | 0.12 | 0.20 | 0.0024 | 0.2641 | 0 | MSX2 |
| chr5:174151479-174152364 | 0.34 | 0.12 | 0.21 | 0.0024 | 0.2803 | 0 | MSX2 | chr1:61508643-61509282 | 0.27 | 0.07 | 0.20 | 0.0005 | 0.1721 | 33663 | NFIA |
| chr5:72746990-72747587 | 0.31 | 0.10 | 0.21 | 0.0008 | 0.2318 | 46662 | BTF3 | chr1:65991002-65991811 | 0.30 | 0.10 | 0.20 | 0.0059 | 0.2811 | 0 | LEPR |
| chr1:61508643-61509282 | 0.30 | 0.08 | 0.21 | 0.0006 | 0.2161 | 33663 | NFIA | chr19:5827755-5828405 | 0.22 | 0.02 | 0.20 | 0.0001 | 0.1000 | 0 | NRTN |
| chr11:30038523-30038823 | 0.33 | 0.12 | 0.21 | 0.0021 | 0.2803 | 0 | KCNA4 | chr11:30038523-30038823 | 0.31 | 0.12 | 0.20 | 0.0037 | 0.2706 | 0 | KCNA4 |
| chr5:16179065-16180420 | 0.30 | 0.09 | 0.21 | 0.0040 | 0.2933 | 0 | MARCH11 | chr4:164252955-164253471 | 0.27 | 0.07 | 0.19 | 0.0056 | 0.2811 | 0 | NPY1R |
| chr7:32467435-32467948 | 0.34 | 0.14 | 0.21 | 0.0036 | 0.2927 | 28561 | LOC100130673 | chr5:72746990-72747587 | 0.28 | 0.08 | 0.19 | 0.0009 | 0.2062 | 46662 | BTF3 |
| chr14:85997469-85998637 | 0.28 | 0.08 | 0.21 | 0.0020 | 0.2803 | 0 | FLRT2 | chr2:176993480-176995557 | 0.25 | 0.06 | 0.19 | 0.0014 | 0.2435 | 0 | HOXD8 |
| chr19:5827755-5828405 | 0.23 | 0.03 | 0.20 | 0.0001 | 0.1151 | 0 | NRTN | chr14:85997469-85998637 | 0.26 | 0.07 | 0.19 | 0.0018 | 0.2599 | 0 | FLRT2 |
| chr2:176993480-176995557 | 0.26 | 0.06 | 0.20 | 0.0013 | 0.2518 | 0 | HOXD8 | chr11:132952539-132953307 | 0.28 | 0.09 | 0.19 | 0.0010 | 0.2247 | 0 | OPCML |
| chr20:11871375-11872207 | 0.26 | 0.06 | 0.20 | 0.0050 | 0.3018 | 0 | BTBD3 | chr9:79630969-79631749 | 0.31 | 0.12 | 0.19 | 0.0024 | 0.2641 | 2821 | FOXB2 |
| chr1:65991002-65991811 | 0.31 | 0.11 | 0.20 | 0.0065 | 0.3057 | 0 | LEPR | chr17:15686219-15686474 | 0.31 | 0.13 | 0.19 | 0.0031 | 0.2684 | 3689 | MEIS3P1 |
| chr6:1378446-1379318 | 0.24 | 0.04 | 0.20 | 0.0039 | 0.2933 | 10750 | FOXF2 | chr15:100880958-100882438 | 0.23 | 0.05 | 0.18 | 0.0002 | 0.1178 | 0 | ADAMTS17 |
| chr2:119606039-119606313 | 0.32 | 0.12 | 0.20 | 0.0029 | 0.2834 | 279 | EN1 | chr2:119606039-119606313 | 0.29 | 0.11 | 0.18 | 0.0032 | 0.2687 | 279 | EN1 |
| chr11:132952539-132953307 | 0.31 | 0.11 | 0.20 | 0.0019 | 0.2803 | 0 | OPCML | chr9:98273679-98273908 | 0.25 | 0.07 | 0.18 | 0.0020 | 0.2635 | 0 | PTCH1 |
| chr9:79630969-79631749 | 0.32 | 0.13 | 0.20 | 0.0023 | 0.2803 | 2821 | FOXB2 | chr8:106330519-106332120 | 0.25 | 0.07 | 0.18 | 0.0014 | 0.2435 | 0 | ZFPM2 |
| chr8:106330519-106332120 | 0.27 | 0.08 | 0.20 | 0.0013 | 0.2518 | 0 | ZFPM2 | chr5:175223610-175224679 | 0.24 | 0.06 | 0.18 | 0.0028 | 0.2684 | 0 | CPLX2 |
| chr15:28753440-28753940 | 0.31 | 0.11 | 0.19 | 0.0026 | 0.2803 | 10816 | GOLGA8G | chr4:7940564-7941853 | 0.28 | 0.11 | 0.18 | 0.0012 | 0.2421 | 0 | AFAP1 |
| chr15:100880958-100882438 | 0.24 | 0.05 | 0.19 | 0.0002 | 0.1464 | 0 | ADAMTS17 | chr10:100992157-100992687 | 0.26 | 0.08 | 0.18 | 0.0007 | 0.1917 | 0 | HPSE2 |
| chr4:4873263-4873613 | 0.28 | 0.09 | 0.19 | 0.0008 | 0.2318 | 7602 | MSX1 | chr4:128544032-128544903 | 0.27 | 0.09 | 0.18 | 0.0046 | 0.2718 | 9183 | INTU |
| chr17:15686219-15686474 | 0.32 | 0.13 | 0.19 | 0.0027 | 0.2805 | 3689 | MEIS3P1 | chr4:118006539-118006859 | 0.22 | 0.05 | 0.17 | 0.0020 | 0.2641 | 0 | TRAM1L1 |
| chr2:130737206-130737718 | 0.26 | 0.07 | 0.19 | 0.0053 | 0.3057 | 0 | RAB6C-AS1 | chr1:236227683-236228817 | 0.24 | 0.06 | 0.17 | 0.0050 | 0.2754 | 0 | NID1 |
| chr9:98273679-98273908 | 0.27 | 0.08 | 0.19 | 0.0029 | 0.2834 | 0 | PTCH1 | chr20:55500348-55501102 | 0.25 | 0.07 | 0.17 | 0.0035 | 0.2706 | 242706 | BMP7 |
| chr10:100992157-100992687 | 0.26 | 0.08 | 0.19 | 0.0009 | 0.2379 | 0 | HPSE2 | chr4:4873263-4873613 | 0.26 | 0.09 | 0.17 | 0.0011 | 0.2347 | 7602 | MSX1 |
| chr4:7940564-7941853 | 0.30 | 0.11 | 0.18 | 0.0011 | 0.2505 | 0 | AFAP1 | chr4:55092962-55093242 | 0.21 | 0.04 | 0.17 | 0.0047 | 0.2727 | 0 | PDGFRA |
| chr4:128544032-128544903 | 0.28 | 0.10 | 0.18 | 0.0043 | 0.2933 | 9183 | INTU | chr19:54483022-54483572 | 0.21 | 0.04 | 0.17 | 0.0013 | 0.2432 | 0 | CACNG8 |
| chr20:55500348-55501102 | 0.27 | 0.08 | 0.18 | 0.0037 | 0.2933 | 242706 | BMP7 | chr2:183902403-183903625 | 0.23 | 0.06 | 0.17 | 0.0005 | 0.1681 | 0 | NCKAP1 |
| chr5:175223610-175224679 | 0.25 | 0.07 | 0.18 | 0.0033 | 0.2861 | 0 | CPLX2 | chr19:54393040-54393300 | 0.26 | 0.09 | 0.17 | 0.0052 | 0.2786 | 0 | PRKCG |
| chr2:183902403-183903625 | 0.25 | 0.07 | 0.18 | 0.0003 | 0.1548 | 0 | NCKAP1 | chr5:87437096-87437505 | 0.23 | 0.06 | 0.17 | 0.0014 | 0.2432 | 53517 | TMEM161B |
| chr5:87437096-87437505 | 0.26 | 0.08 | 0.18 | 0.0024 | 0.2803 | 53517 | TMEM161B | chr19:31839636-31843049 | 0.24 | 0.07 | 0.17 | 0.0022 | 0.2641 | 0 | TSHZ3 |
| chr1:236227683-236228817 | 0.25 | 0.07 | 0.18 | 0.0042 | 0.2933 | 0 | NID1 | chr1:152487979-152488270 | 0.25 | 0.08 | 0.17 | 0.0059 | 0.2811 | 0 | CRCT1 |
| chr4:164252955-164253471 | 0.26 | 0.08 | 0.18 | 0.0044 | 0.2933 | 0 | NPY1R | chr18:55094826-55096310 | 0.27 | 0.10 | 0.17 | 0.0052 | 0.2786 | 6606 | ONECUT2 |
| chr11:12398966-12399863 | 0.27 | 0.09 | 0.17 | 0.0018 | 0.2803 | 0 | PARVA | chr11:12398966-12399863 | 0.25 | 0.08 | 0.17 | 0.0014 | 0.2435 | 0 | PARVA |
| chr6:19837505-19839314 | 0.28 | 0.11 | 0.17 | 0.0025 | 0.2803 | 0 | ID4 | chr6:19837505-19839314 | 0.26 | 0.10 | 0.16 | 0.0030 | 0.2684 | 0 | ID4 |
| chr4:55092962-55093242 | 0.22 | 0.05 | 0.17 | 0.0063 | 0.3057 | 0 | PDGFRA | chr9:1042418-1042973 | 0.25 | 0.09 | 0.16 | 0.0028 | 0.2684 | 7372 | DMRT2 |
| chr19:54483022-54483572 | 0.21 | 0.04 | 0.17 | 0.0016 | 0.2746 | 0 | CACNG8 | chr2:223161532-223161919 | 0.20 | 0.04 | 0.16 | 0.0015 | 0.2435 | 0 | PAX3 |
| chr18:55094826-55096310 | 0.29 | 0.11 | 0.17 | 0.0062 | 0.3057 | 6606 | ONECUT2 | chr16:54962423-54967805 | 0.25 | 0.09 | 0.16 | 0.0031 | 0.2684 | 0 | CRNDE |
| chr19:31839636-31843049 | 0.25 | 0.08 | 0.17 | 0.0022 | 0.2803 | 0 | TSHZ3 | chr1:94702691-94703344 | 0.23 | 0.07 | 0.16 | 0.0031 | 0.2684 | 0 | ARHGAP29 |
| chr4:118006539-118006859 | 0.23 | 0.05 | 0.17 | 0.0024 | 0.2803 | 0 | TRAM1L1 | chr2:45511-46559 | 0.24 | 0.08 | 0.16 | 0.0023 | 0.2641 | 0 | FAM110C |
| chr9:1042418-1042973 | 0.27 | 0.10 | 0.17 | 0.0031 | 0.2834 | 7372 | DMRT2 | chr7:27203916-27206462 | 0.23 | 0.07 | 0.16 | 0.0046 | 0.2718 | 0 | HOXA10-HOXA9 |
| chr18:75611919-75612142 | 0.20 | 0.03 | 0.17 | 0.0002 | 0.1151 | 629822 | GALR1 | chr8:41165853-41167140 | 0.24 | 0.08 | 0.16 | 0.0043 | 0.2718 | 0 | SFRP1 |
| chr2:45511-46559 | 0.26 | 0.09 | 0.17 | 0.0012 | 0.2518 | 0 | FAM110C | chr1:50489418-50489846 | 0.24 | 0.08 | 0.16 | 0.0042 | 0.2718 | 0 | AGBL4 |
| chr8:41165853-41167140 | 0.26 | 0.09 | 0.17 | 0.0037 | 0.2933 | 0 | SFRP1 | chr20:48098513-48099560 | 0.30 | 0.14 | 0.15 | 0.0052 | 0.2786 | 0 | KCNB1 |
| chr16:54962423-54967805 | 0.27 | 0.10 | 0.17 | 0.0029 | 0.2834 | 0 | CRNDE | chr7:20817456-20818227 | 0.24 | 0.08 | 0.15 | 0.0028 | 0.2684 | 3666 | SP8 |
| chr3:45187027-45187946 | 0.23 | 0.06 | 0.17 | 0.0060 | 0.3057 | 0 | CDCP1 | chr4:147576110-147576762 | 0.21 | 0.06 | 0.15 | 0.0067 | 0.2848 | 12486 | POU4F2 |
| chr15:26327496-26327896 | 0.22 | 0.05 | 0.17 | 0.0002 | 0.1464 | 29228 | LINC02346 | chr18:75611919-75612142 | 0.18 | 0.03 | 0.15 | 0.0002 | 0.1158 | 629822 | GALR1 |
| chr7:27212417-27214396 | 0.26 | 0.09 | 0.17 | 0.0030 | 0.2834 | 0 | HOXA10-HOXA9 | chr3:145878431-145879287 | 0.22 | 0.06 | 0.15 | 0.0039 | 0.2718 | 0 | PLOD2 |
| chr7:27203916-27206462 | 0.25 | 0.08 | 0.17 | 0.0039 | 0.2933 | 0 | HOXA10-HOXA9 | chr13:53419898-53422872 | 0.26 | 0.11 | 0.15 | 0.0044 | 0.2718 | 0 | PCDH8 |
| chr1:91183241-91184540 | 0.24 | 0.07 | 0.17 | 0.0058 | 0.3057 | 446 | BARHL2 | chr20:590223-591222 | 0.25 | 0.10 | 0.15 | 0.0046 | 0.2718 | 0 | TCF15 |
| chr20:4228533-4230496 | 0.31 | 0.15 | 0.16 | 0.0027 | 0.2805 | 0 | ADRA1D | chr4:85503547-85504893 | 0.19 | 0.04 | 0.15 | 0.0036 | 0.2706 | 0 | CDS1 |
| chr2:223161532-223161919 | 0.22 | 0.05 | 0.16 | 0.0016 | 0.2746 | 0 | PAX3 | chr12:59313560-59314452 | 0.20 | 0.05 | 0.15 | 0.0026 | 0.2671 | 0 | LRIG3 |
| chr1:94702691-94703344 | 0.24 | 0.08 | 0.16 | 0.0034 | 0.2861 | 0 | ARHGAP29 | chr2:177052958-177054350 | 0.24 | 0.09 | 0.15 | 0.0060 | 0.2811 | 0 | HAGLR |
| chr5:35617856-35618339 | 0.20 | 0.04 | 0.16 | 0.0003 | 0.1536 | 0 | SPEF2 | chr5:35617856-35618339 | 0.19 | 0.04 | 0.15 | 0.0005 | 0.1655 | 0 | SPEF2 |
| chr12:59313560-59314452 | 0.22 | 0.06 | 0.16 | 0.0031 | 0.2834 | 0 | LRIG3 | chr18:73167403-73167920 | 0.27 | 0.12 | 0.15 | 0.0057 | 0.2811 | 27813 | SMIM21 |
| chr1:50489418-50489846 | 0.25 | 0.09 | 0.16 | 0.0049 | 0.2994 | 0 | AGBL4 | chr19:51227662-51228883 | 0.22 | 0.07 | 0.15 | 0.0014 | 0.2432 | 0 | CLEC11A |
| chr19:51227662-51228883 | 0.24 | 0.08 | 0.16 | 0.0017 | 0.2803 | 0 | CLEC11A | chr7:27212417-27214396 | 0.23 | 0.08 | 0.15 | 0.0038 | 0.2706 | 0 | HOXA10-HOXA9 |
| chr3:145878431-145879287 | 0.23 | 0.07 | 0.16 | 0.0038 | 0.2933 | 0 | PLOD2 | chr1:48937305-48937683 | 0.20 | 0.05 | 0.15 | 0.0018 | 0.2599 | 0 | SPATA6 |
| chr2:177052958-177054350 | 0.26 | 0.10 | 0.16 | 0.0038 | 0.2933 | 0 | HAGLR | chr3:39851026-39851820 | 0.18 | 0.03 | 0.15 | 0.0014 | 0.2435 | 0 | MYRIP |
| chr19:54409967-54410200 | 0.20 | 0.04 | 0.16 | 0.0046 | 0.2933 | 0 | PRKCG | chr20:4228533-4230496 | 0.30 | 0.15 | 0.15 | 0.0054 | 0.2801 | 0 | ADRA1D |
| chr18:73167403-73167920 | 0.28 | 0.13 | 0.16 | 0.0055 | 0.3057 | 27813 | SMIM21 | chr15:96873409-96877721 | 0.28 | 0.13 | 0.15 | 0.0053 | 0.2786 | 0 | NR2F2 |
| chr2:132121264-132121762 | 0.20 | 0.04 | 0.16 | 0.0034 | 0.2861 | 0 | RAB6D | chr10:118927022-118928132 | 0.23 | 0.08 | 0.15 | 0.0042 | 0.2718 | 0 | MIR3663 |
| chr4:85503547-85504893 | 0.20 | 0.04 | 0.16 | 0.0027 | 0.2805 | 0 | CDS1 | chr6:80656745-80657593 | 0.19 | 0.04 | 0.15 | 0.0004 | 0.1400 | 0 | ELOVL4 |
| chr20:590223-591222 | 0.26 | 0.11 | 0.16 | 0.0045 | 0.2933 | 0 | TCF15 | chr12:88973461-88974666 | 0.21 | 0.07 | 0.15 | 0.0053 | 0.2786 | 0 | KITLG |
| chr7:20817456-20818227 | 0.26 | 0.10 | 0.16 | 0.0036 | 0.2899 | 3666 | SP8 | chr7:28995306-28998541 | 0.25 | 0.10 | 0.15 | 0.0026 | 0.2672 | 0 | TRIL |
| chr10:47008085-47008410 | 0.22 | 0.07 | 0.16 | 0.0033 | 0.2861 | 3345 | ANXA8 | chr2:132121264-132121762 | 0.19 | 0.04 | 0.15 | 0.0046 | 0.2718 | 0 | RAB6D |
| chr8:24812947-24814299 | 0.24 | 0.08 | 0.16 | 0.0038 | 0.2933 | 0 | NEFL | chr16:31580560-31581023 | 0.19 | 0.04 | 0.15 | 0.0051 | 0.2764 | 0 | YBX3P1 |
| chr10:8091375-8098329 | 0.22 | 0.07 | 0.16 | 0.0018 | 0.2803 | 0 | GATA3-AS1 | chr14:85999533-86000478 | 0.22 | 0.08 | 0.15 | 0.0044 | 0.2718 | 0 | FLRT2 |
| chr7:28995306-28998541 | 0.27 | 0.11 | 0.16 | 0.0025 | 0.2803 | 0 | TRIL | chr10:47008085-47008410 | 0.20 | 0.06 | 0.15 | 0.0031 | 0.2684 | 3345 | ANXA8 |
| chr12:88973461-88974666 | 0.24 | 0.08 | 0.16 | 0.0056 | 0.3057 | 0 | KITLG | chr12:20521617-20523122 | 0.23 | 0.09 | 0.15 | 0.0039 | 0.2718 | 0 | PDE3A |
| chr3:39851026-39851820 | 0.19 | 0.04 | 0.16 | 0.0015 | 0.2746 | 0 | MYRIP | chr2:211089414-211090176 | 0.20 | 0.05 | 0.15 | 0.0027 | 0.2684 | 0 | ACADL |
| chr12:20521617-20523122 | 0.26 | 0.10 | 0.16 | 0.0042 | 0.2933 | 0 | PDE3A | chr1:65775019-65775746 | 0.19 | 0.05 | 0.15 | 0.0036 | 0.2706 | 0 | DNAJC6 |
| chr10:118927022-118928132 | 0.25 | 0.09 | 0.15 | 0.0048 | 0.2994 | 0 | MIR3663 | chr8:72468561-72469561 | 0.23 | 0.08 | 0.15 | 0.0066 | 0.2848 | 194093 | EYA1 |
| chr2:211089414-211090176 | 0.22 | 0.06 | 0.15 | 0.0022 | 0.2803 | 0 | ACADL | chr7:79081566-79081879 | 0.24 | 0.09 | 0.15 | 0.0067 | 0.2848 | 0 | MAGI2 |
| chr13:53419898-53422872 | 0.27 | 0.11 | 0.15 | 0.0045 | 0.2933 | 0 | PCDH8 | chr6:39692744-39692966 | 0.18 | 0.04 | 0.15 | 0.0043 | 0.2718 | 0 | KIF6 |
| chr20:8112885-8113592 | 0.21 | 0.05 | 0.15 | 0.0016 | 0.2803 | 0 | PLCB1 | chr10:8091375-8098329 | 0.21 | 0.06 | 0.14 | 0.0021 | 0.2641 | 0 | GATA3-AS1 |
| chr6:80656745-80657593 | 0.20 | 0.04 | 0.15 | 0.0004 | 0.1652 | 0 | ELOVL4 | chr20:8112885-8113592 | 0.19 | 0.05 | 0.14 | 0.0017 | 0.2594 | 0 | PLCB1 |
| chr8:72468561-72469561 | 0.24 | 0.09 | 0.15 | 0.0051 | 0.3044 | 194093 | EYA1 | chr8:24812947-24814299 | 0.21 | 0.07 | 0.14 | 0.0044 | 0.2718 | 0 | NEFL |
| chr8:12990091-12990914 | 0.20 | 0.05 | 0.15 | 0.0039 | 0.2933 | 0 | DLC1 | chr22:17083385-17083628 | 0.18 | 0.04 | 0.14 | 0.0047 | 0.2727 | 0 | TPTEP1 |
| chr7:79081566-79081879 | 0.26 | 0.11 | 0.15 | 0.0065 | 0.3057 | 0 | MAGI2 | chr6:125283125-125284389 | 0.20 | 0.06 | 0.14 | 0.0056 | 0.2811 | 0 | RNF217-AS1 |
| chr1:27961560-27961810 | 0.15 | 0.37 | -0.22 | 3.52E-06 | 0.015163 | 0 | FGR | chr5:139040820-139041028 | 0.13 | 0.36 | -0.22 | 4.37E-06 | 0.010331 | 0 | CXXC5 |
| chr5:139040820-139041028 | 0.15 | 0.40 | -0.25 | 4.52E-06 | 0.016705 | 0 | CXXC5 | chr1:27961560-27961810 | 0.14 | 0.36 | -0.22 | 2.66E-06 | 0.009826 | 0 | FGR |
| chr11:47399789-47400006 | 0.16 | 0.41 | -0.25 | 3.26E-06 | 0.015163 | 0 | SLC39A13 | chr11:47399789-47400006 | 0.13 | 0.36 | -0.23 | 1.59E-06 | 0.008215 | 0 | SLC39A13 |

adj.: adjusted; CLL: chronic lymphocytic leukemia; Comb.: combined; CTRL: controls.

Covariate adjustment refer to adjustment for lymphocyte counts as described in Material and Methods.
